# Volatile Organic Compound Assessment as a Screening Tool for Early Detection of Gastrointestinal Diseases

**DOI:** 10.3390/microorganisms11071822

**Published:** 2023-07-17

**Authors:** Costa Dalis, Fikir M. Mesfin, Krishna Manohar, Jianyun Liu, W. Christopher Shelley, John P. Brokaw, Troy A. Markel

**Affiliations:** Department of Surgery, Indiana University School of Medicine, Indianapolis, IN 46202, USA

**Keywords:** electronic nose, dysbiosis, volatile organic compound, gastrointestinal diseases

## Abstract

Gastrointestinal (GI) diseases have a high prevalence throughout the United States. Screening and diagnostic modalities are often expensive and invasive, and therefore, people do not utilize them effectively. Lack of proper screening and diagnostic assessment may lead to delays in diagnosis, more advanced disease at the time of diagnosis, and higher morbidity and mortality rates. Research on the intestinal microbiome has demonstrated that dysbiosis, or unfavorable alteration of organismal composition, precedes the onset of clinical symptoms for various GI diseases. GI disease diagnostic research has led to a shift towards non-invasive methods for GI screening, including chemical-detection tests that measure changes in volatile organic compounds (VOCs), which are the byproducts of bacterial metabolism that result in the distinct smell of stool. Many of these tools are expensive, immobile benchtop instruments that require highly trained individuals to interpret the results. These attributes make them difficult to implement in clinical settings. Alternatively, electronic noses (E-noses) are relatively cheaper, handheld devices that utilize multi-sensor arrays and pattern recognition technology to analyze VOCs. The purpose of this review is to (1) highlight how dysbiosis impacts intestinal diseases and how VOC metabolites can be utilized to detect alterations in the microbiome, (2) summarize the available VOC analytical platforms that can be used to detect aberrancies in intestinal health, (3) define the current technological advancements and limitations of E-nose technology, and finally, (4) review the literature surrounding several intestinal diseases in which headspace VOCs can be used to detect or predict disease.

## 1. Introduction

Gastrointestinal (GI) diseases have a high prevalence throughout the United States. Screening and diagnostic modalities are often expensive and invasive. Lack of proper screening and diagnostic assessment may lead to delays in diagnosis, more advanced disease at the time of diagnosis, and higher morbidity and mortality rates [1]. Volatile organic compound (VOC) profiling may be advantageous as non-invasive methods to assess intestinal health. VOCs are a diverse group of compounds that are emitted from many human sources, including the breath, skin, GI tract, urine, and others, and are formed as a byproduct from both eukaryotic and prokaryotic cell metabolism. VOCs can be assessed by multiple analytical platforms, but most are large, cumbersome machines that require specialized training. Therefore, the use of electronic noses (E-noses), which are a subtype of electronic-sensing technology designed to reproduce human smell via sensor arrays and pattern recognition algorithms, may be more clinically applicable as they are often potable machines that can be used at the bedside. The purpose of this review is to (1) highlight how dysbiosis impacts intestinal diseases and how VOC metabolites can be utilized to detect alterations in the intestinal microbiome, (2) summarize the different VOC analytical platforms that can be used to detect aberrancies in intestinal health, (3) define the current technological advancements and limitations of E-nose technology, and finally, (4) review the literature surrounding several intestinal diseases in which headspace VOCs can be used to detect or predict disease.

### 1.1. Dysbiosis and the Production of VOCs

The gut microbiome is vital for many functions, some of which include immune system homeostasis, host energy metabolism, and maintaining the integrity of the entire GI tract. Unfavorable alterations in the composition of the microbiome, referred to as dysbiosis, have been linked to many GI illnesses along with extraintestinal detrimental health consequences, including type I diabetes mellitus, autoimmune-mediated arthritis, encephalomyelitis, and others [2]. The microbiome has been estimated to contain approximately 10^14^ microorganisms, most of them being bacterial species, such as *Bacteroidetes* and *Firmicutes* [3,4]. A vital element for a healthy gut microbiome is diversity, which can be lost from several influences, including poor diet, immune dysregulation, inflammation, and antibiotic use. Bacteria in the gut ferment non-starch polysaccharides as a means of metabolism during their lifecycle, producing an odorous gas composed of VOCs, which are vaporous, carbon-based molecules at environmental temperature. Not only are VOCs found in the intestine, but also have been shown to be emitted from the skin, breath, feces, and urine. Research has demonstrated that certain bacterial species produce specific VOCs. For example, the *Bacteroidetes* species has been shown to produce ethanoic, propionic, butanoic, pentanoic, and hexanoic acids [5]. While fecal VOCs only differ slightly between individuals, the overall profile can be very different between health and disease, reflecting how VOCs can be used as specific biomarkers of intestinal diseases via altered microbial metabolic activity. Therefore, the presence of differing fecal VOC profiles is the result of altered gut microbial fermentation, and has the potential to be a surrogate marker for intestinal dysbiosis and disease [3].

The intracellular molecular signaling pathways associated with dysbiosis have largely been associated with activation of pattern recognition receptors (PRRs). PRRs are pro-inflammatory transmembrane proteins that can recognize different patterns released during pathological states [6]. For example, damage-associated molecular patterns (DAMPs) are released from dying cells and cells undergoing stress, while pathogen-associated molecular patterns (PAMPs) are released from various microbes, and include substances such as lipopolysaccharide (LPS), lipoteichoic acid, and peptidoglycan. PRRs are found in a variety of cells throughout the GI tract, including intestinal epithelial cells, neutrophils, fibroblasts, and antigen-presenting cells, suggesting that a large inflammatory and immune response can be mounted during a pathological state [7].

**Figure 1:**
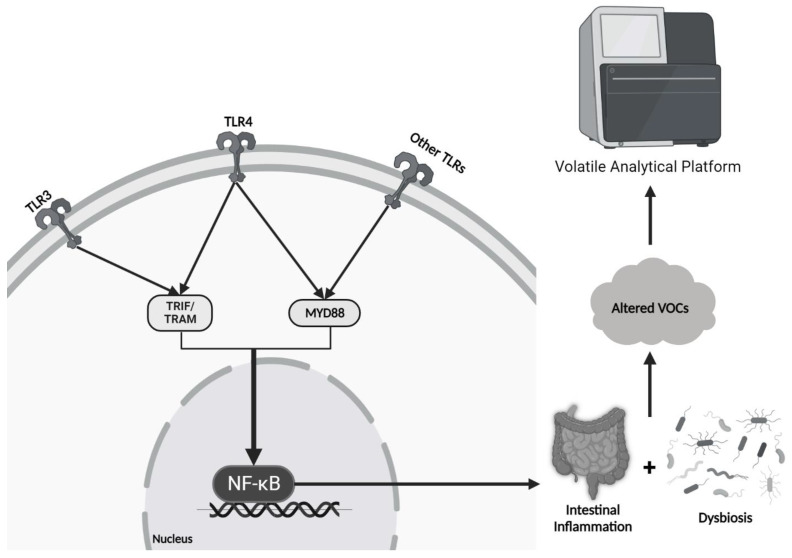
**Signaling Pathway of Dysbiosis and the Production of Altered VOCs:** Inflammatory mediators from pathologic bacteria signal through TLR and NF-kB signaling cascades to further promote inflammation and ongoing dysbiosis. Changes in bacterial composition promote altered volatile organic compounds in the stool, and hence, an altered smell and different signals detected by the E-nose.

DAMPs and PAMPs typically signal through Toll-like receptors (TLRs). There are two intracellular signaling pathways downstream of TLR activation, namely the MyD88-dependent or MyD88-independent signaling pathway. MyD88 is an adapter protein that induces nuclear factor kappa light chain enhancer of activated B cells (NF-κB) gene transcription, which promotes the synthesis of many pro-inflammatory cytokines. Virtually all TLRs, except TLR3, function through the MyD88-dependent pathway. Activation of the MyD88-independent tripartite motif TRIF/TRAM pathway, majorly through TLR3 and minorly through TLR4, leads to the synthesis of type I interferons, such as IFN-β, which can promote NF-κB signaling [8] (Figure 1).

In a normal physiological state, there is always a small amount of GI inflammation occurring due to constant binding of PAMPs and DAMPs to PRRs as the body defends against pathogens and is exposed to various stressors [9]. However, it is thought that the gut microbiota play a vital role in suppressing NF-κB activation, resulting in attenuation of inflammation and restoration of a healthy balance of pro- and anti-inflammatory processes [10]. Therefore, in the setting of dysbiosis, NF-κB signaling suppression is lost, resulting in excessive inflammation with subsequent tissue damage. Microbial metabolic activity is altered when subjected to this type of stress, resulting in differential VOC release, which can be detected using volatile analytical platforms (e.g., E-nose) (Figure 1).

### 1.2. Traditional Analytical Platforms to Assess VOCs

Multiple analytical platforms exist to assess volatile organic compounds (Table 1). Gas chromatography mass spectrometry (GC-MS) is a technique that separates individual components based on volatility and mass from a solid or liquid sample source, allowing for analysis of purity and quantity [11]. First, the sample of interest is placed into the injection port, where it is heated until vaporized into the gaseous phase and is passed through a capillary column with the help of an inert carrier gas; this process is referred to as the “mobile phase”. This column is coated to provide a surface for compounds in the sample to interact and slow their movement so proper separation can take place; this process is referred to as the “stationary phase”. Upon reaching the end of the column, the compounds are hit with 70 electron volts, which causes them to break into cations. The cations then can travel through an electromagnetic field that filters them based on their mass. Finally, the detector amplifies and quantifies the number of ions associated with the specific mass of the fragment, generating a mass/charge (*m/z*) ratio [11]. This information is transferred to a computer where a mass spectrum is created and proper identification and/or purity of the components of the sample can be accomplished. To decrease the loss of VOCs into the atmosphere, GC-MS can also be coupled to solid-phase microextraction (SPME), in which absorptive fibers are used to trap VOCs directly from the headspace for subsequent desorption and analysis, thereby skipping the liquid phase seen in traditional GC-MS [12].

Field asymmetric ion mobility spectrometry (FAIMS) is an ion mobility spectrometry (IMS) technique performed at atmospheric pressure that can separate ions based on their behavior in both strong and weak electric fields [13]. The electrospray emitter contains the solution of interest and is responsible for the production of gaseous phase ions that are emitted into the FAIMS interface. Inside the interface, an asymmetric electric field (the dispersion voltage) is applied to the inner cylindrical electrode to separate ions as they traverse the interface in a flow of nitrogen. This asymmetric electric field is what differentiates FAIMS from traditional IMS. Spatial dispersion is achieved by applying a high field for a short duration, which pulls cations towards the inner electrode, then switching to a low field of opposite polarity for twice the duration, which pushes the cations back toward their original trajectories. For an individual ion, this voltage switching pattern is repeated thousands of times, with each cycle causing greater dispersion [14]. Only cations with a stable flight path exit into the mass spectrometer for detection and sequencing, which can be selected by applying a secondary direct current potential (the compensation voltage) to the inner electrode.

Selected ion flow tube mass spectrometry (SIFT-MS) has the ability to quantify compounds in air to less than one parts per thousand [15]. First, a mixture of air and water vapor is passed through a microwave discharge composed of ionized gases such as NO^+^, O_2_^+^, and H_3_O^+^, which are subsequently focused into a quadrupole mass filter. Inside the filter, the ions are separated based on their *m*/*z* ratio, and every few milliseconds, the quadrupole selects a different reagent ion to enter the flow tube, which consists of a continuous stream of the sample gas of interest and carrier gases (e.g., helium or nitrogen). The reagent ions and molecules from the sample gas react with each other to yield stable and predictable ionization products, which are focused into a second quadrupole and finally transmitted to a detector for sequencing [15]. Proton transfer reaction mass spectrometry (PTR-MS) functions similarly to SIFT-MS in that protonation is required for ionization of gases; however, PTR-MS utilizes H_3_O^+^ as a primary ion source and not NO^+^ or O_2_^+^ [16]. This difference provides an advantage as most ambient gases found in mixtures (e.g., N_2_, O_2_, and CO_2_) have a lower proton affinity than water, and will therefore not disrupt the ionization process.

### 1.3. Biosensors

Biosensors are another modality that is of increasing interest in disease screening and diagnosis. Biosensors are compact devices that measure specific biomarkers from samples taken from the body (e.g., blood and interstitial fluid). Some biosensors are wearable (e.g., glucose sensors used in diabetes monitoring) and provide continuous measurements [17]. The most notable advantage seen with biosensors is the immediate results they provide, but additionally they are portable, user friendly, and cost-effective, enabling self-management for patients and avoidance of unnecessary healthcare visits [18]. The four main components of a biosensor include a molecular recognition process, a signal generator (typically optical, electronic, or magnetic), a disposable sensor device, and a reader instrument [19]. Traditional molecules that biosensors recognize include antibodies, aptamers, and enzymes, however, novel research has expanded their use to include detection of VOCs, ensuing a potential future of their use to detect GI and other VOC-based pathologies similar to that of chemical-detection tests and E-nose technologies [20,21,22,23,24,25].

### 1.4. Electronic Noses

In 1988, Gardner and Bartlett coined the term “electronic nose” and later defined it as “an instrument which comprises an array of electronic chemical sensors with partial specificity and appropriate pattern recognition system, capable of recognizing simple or complex odors” [26]. E-noses are a subtype of electronic-sensing technology designed to reproduce human smell via sensor arrays and pattern recognition algorithms, where gaseous molecular signals can be transduced into electrical ones [27]. While there are many variations of E-noses, they all operate utilizing three main parts: (1) a sample delivery, where the E-nose is exposed to the smell of interest for pretraining purposes, (2) a detection system that recognizes a physical change in the sensor caused by adsorption of gaseous compounds on the sensor’s surface, and (3) machine learning algorithms that analyze the detected information via reference library databases to provide a pattern recognition output that describes the odor/aroma of interest [28].

E-noses are portable, provide rapid results, are inexpensive compared to chemical-detection tests, and are easy to operate, making them more favorable to use in a clinical or hospital setting [29]. E-noses were originally developed in the early 1980s to allow for accurate, on-the-spot discrimination of odors that humans cannot sense in a cost-effective manner. This has led to a rapid expansion of their use in agriculture and forestry, industrial processes, environmental pollutant analysis, food and beverage inspection, healthcare, and many others [27,30]. Indeed, the market potential for E-noses is growing, as it is estimated that the global electronic nose market is expected to register a compound annual growth rate of 11.3% between 2023 and 2028 and nearly doubling its market value between 2022 and 2030 from approximately USD 42.4 billion to USD 81 billion [31]. While the market potential is growing, the commercialization of E-noses is likely to experience obstacles. Due to the intricate network of sensors that E-noses utilize, they can range in prices from about USD 9000 to USD 150,000 which makes many potential consumers unable to purchase commercial E-nose products [32]. Until a less expensive method of producing E-noses is standardized, their use will likely be limited to larger corporations.

**Figure 2:**
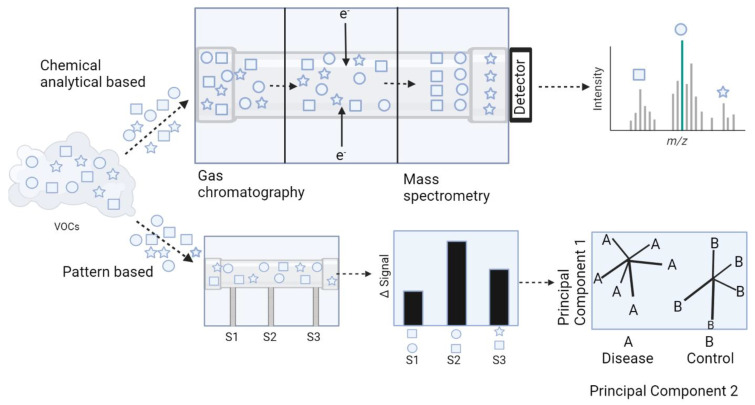
**Comparison of VOC detection and graphical output using GC-MS and E-nose technology.** Chemical analytical based modalities for VOC detection utilize mass spectrometry as a means of identification of unique biomarkers and generate mass spectra as an output for data interpretation. Alternatively, E-noses recognize patterns of VOCs and generate PCA plots for data interpretation.

Principal component analysis (PCA) is the primary method used by E-noses to graphically depict the patterns they recognize. Although other methods exist, including linear discriminant analysis (LDA), discriminant function analysis (DFA), cluster analysis, and partial least squares [33], PCA is the typical output for E-noses as compared to mass to charge ratios depicted in traditional analytical VOC platforms (Figure 2). PCA is a multivariate technique in which new orthogonal variables (called principal components) are extrapolated from inter-correlated, dependent variables from a data set and are displayed as patterns of similarity as points on a map [34]. The greater the distance between the principal components, the more different their patterns are, which is often the case when comparing VOC profiles between healthy and diseased individuals.

E-noses can vary in the type of sensor used. Each type of sensor utilizes a unique detection principle and is sensitive to specific types of material. Given this, each sensor also comes with advantages and disadvantages, such as response and recovery times, sensitivities, physical size, and range of detection [27]. The types of sensors that have been studied regarding GI disease screening and diagnosis include conducting polymer, metal oxide sensors (MOXs), optical, electrochemical (EC), and quartz crystal microbalance (QMB) (Table 2).

Conducting polymer sensors detect changes in electrochemical resistance when odorous molecules adsorb onto the sensor’s surface. There are typically three parts of a conducting polymer sensor, including a pair of gold-plated electrodes, a substrate (e.g., silicon), and an organic polymer that coats the sensor and is capable of conduction via a conjugated pi-electron system [27]. The organic polymer is usually synthesized from its corresponding monomer via chemical or electrochemical oxidation, and the most common monomers include polypyrrole, polyaniline and polythiophene [35].

MOXs are usually coated with tin-dioxide (SnO_2_) doped with a small quantity of catalytic metal additives. Similar to conducting polymer sensors, MOXs detect changes in resistance, but through a different mechanism. At high temperatures (300–500 °C), combustion reactions are elicited when gaseous molecules adsorb onto the sensor and react with oxygen species from the SnO_2_ coating particles, resulting in a change in conductivity and subsequently resistance of the sensor. To achieve such temperatures, a platinum-based heater spiral surrounded by a ceramic support tube is often utilized [27]. At lower temperatures, the combustion reaction rates are either too slow or do not occur at all, and consequently metal-oxide sensors require a large amount of power consumption to maintain necessary temperature ranges [36].

Optical sensors operate via light modulation measurement and can detect changes in absorbance, fluorescence, light polarization, optical layer thickness, or colorimetric dye response. The most basic optical sensors implement metalloporphyrins as color-changing indicators to measure absorbance with a photodetector system and a LED upon exposure to gaseous molecules [27]. EC sensors operate through redox reactions of volatile compounds at a catalytic electrode surface, which is usually coated with a hydrophobic membrane composed of a layer of metal [37]. While this provides the advantage of high sensitivity to electrochemically active gases, it makes them relatively insensitive to chemically inert gases (e.g., aromatic hydrocarbons) [38].

The quartz crystal microbalance (QCM) is a biosensor platform containing a mechanical transducer that operates via mass detection, which allows QCM to detect virtually any type of molecule given that mass is an intrinsic property of all substances [39]. Therefore, QCM is a versatile platform for detecting the various types of disease biomarkers. Indeed, QCM has gained significant interest recently in the field of pathogen detection since biomolecules can be detected via a label-free method in a rapid, highly sensitive manner [40]. The initial work evaluating the feasibility of QCM-based sensors in Enoses was conducted by Di Natale et al. [41], in which breath samples were collected from patients with lung cancer, healthy controls (HC), and post-surgical patients, and results yielded 100% detection in the cancer patients, 94% detection in the controls, and 44% in post-surgical patients. Furthermore, it was confirmed that the E-nose could identify eleven unique VOCs that are diagnostic for lung cancer, with a sensitivity and specificity of 71.4% and 91.4%, respectively.

### 1.5. Limitations in E-Nose Technology

Novel work continues to improve E-nose efficacy, specifically surrounding the optimization of machine learning algorithm techniques. These include feature extraction, modeling, and gas sensor drift compensation [42]. Feature extraction is a technique that attempts to preserve wanted signals for proper identification while simultaneously removing unwanted redundancy and noise. Traditionally, manual feature extraction has been the primary method used, where either time or frequency domains are extracted; however, this requires prior knowledge of data processing along with data from the E-nose itself. While this method has sufficed for odor discrimination in many cases, it requires tedious feature selection and requires prior knowledge of gas sensor technology. Alternatively, feature extraction through learning of raw sensing signals using artificial neural networks (ANNs), such as autoencoder and deep belief network, requires only minimal data processing steps and can still predict odors with high accuracy [43,44,45]. ANNs are models composed of units that combine multiple inputs to produce a single output, mimicking how multiple neurons in the brain fire collectively to elicit a single action [46].

The modeling method works in conjunction with feature extraction to ensure the E-nose can effectively use the information it receives for accurate prediction. Traditionally, linear models have been adopted due to their easy implementation [42]. Recently, deep learning models, such as convolution neural networks (CNN) and long short-term memory (LSTM), have been shown to lead to better performances compared to their linear model counterparts [47,48,49,50,51]. With continued development of feature extraction and modeling, the use of E-noses in predicting diseases in healthcare settings will continue to become more reliable.

While improvements have been made for feature extraction and modeling, gas sensor drifting remains a significant challenge for E-noses, given that it negatively affects both signal and feature consistency. Gas sensor drifting is a phenomenon where over time there is a physical change in the sensor’s surface and primarily occurs through natural aging of the sensor and/or ambient influences such as temperature and humidity [42]. Two recent strategies that have been implemented to overcome gas sensor drifting include ensemble learning and domain adaptation learning. Ensemble learning is a technique that combines individual models together to improve stability and predictive power, permitting a higher predictive performance compared to the use of one model. Domain adaptation is a type of transfer learning in which a model with a domain with insufficient data uses the knowledge of a related domain with adequate data to improve overall performance of the model [52,53,54,55]. Neither strategy has resulted in satisfactory results yet, but progress has been made and is likely to continue improving.

Another limitation of E-nose technology is the variability between different sensor types [56]. Multiple different sensor types makes the findings from one sensor type incomparable to the findings of another because each sensor generates its own unique signal response [29]. Research aimed at standardizing signal responses between devices, controlling design variance, and allowing inter-operability between E-noses of the same sensor type has resulted in progress in resolving some of these limitations; however, inter-operability between different sensors has yet to be achieved, greatly limiting the generalizability of E-noses [57]. Additionally, the recent development of multivariate VOC biomarker association leads to an increased risk of data sets that are too all-encompassing, potentially resulting in false associations [58]. Indeed, many of the studies to date have demonstrated great accuracy and performances with E-noses have based their metrics from training sets rather than large validation sets [57]. Until further research is completed using validation sets to allow E-noses to blindly identify compounds and function in an independent setting, as they would in healthcare settings, their clinical application cannot be fully appreciated.

## 2. Review of the Literature

In a review of the literature from 2004 to 2023 of headspace VOC analysis, a total of 35 studies were identified and included after searching PubMed for terms inclusive of “headspace”, “VOC”, or “gastrointestinal disease”. The majority of these studies were able to successfully differentiate diseased states from controls. The following section provides a non-exhaustive list of GI illnesses that have been studied via VOC analysis.

## 3. Detection of Gastrointestinal Illnesses by VOCs

### 3.1. Inflammatory Bowel Disease and Irritable Bowel Syndrome

Inflammatory bowel disease (IBD) is characterized by chronic inflammation of the GI tract with patterns of flare-ups and remissions. IBD can be split into two main subtypes: ulcerative colitis (UC) and Crohn’s disease (CD). While the pathophysiology of IBD is not completely understood, an association with dysbiosis is presumed [59]. Alterations in short-chain fatty acid (SCFA)-producing bacteria such as *Clostridium* and *Faecalibacterium prausnitzzi*, an increase in mucolytic bacteria such as *Ruminococci*, and increasing sulfate-reducing bacteria such as *Desulfovibrio* have been associated with the symptoms of IBD [60]. A study conducted by Walton et al. [61] found increased concentrations of indoles, alcohols, and esters in patients with active CD. Once these patients were appropriately treated, the concentrations decreased to a point that more closely resembled healthy individuals. Additionally, specific VOC biomarkers have been identified that demonstrate promising results for diagnosing and monitoring patients with IBD, namely propan-1-ol and 1-methyl-4-propan-2-ylcyclohexa-1,4-diene [62]. The dysfunctions associated with IBD include decreased energy supply for enterocytes and degradation of the protective mucosal barrier. These aberrancies lead to enterocyte inflammation and/or death and increased bacterial invasion [60]. The most common volatile analytical platforms used in detecting IBD via VOC analysis include GC-MS, FAIMS, SIFT-MS, and E-nose technology (Table 3).

De Meij and colleagues [63] performed VOC analysis on pediatric patients to differentiate between IBD and healthy controls during active and latent disease states using the Cyranose 320^®^, which is a conducting polymer sensor-based E-nose. To accomplish this, a total of 55 children newly diagnosed with IBD (26 with UC and 29 with CD) each provided two stool samples, one at baseline and one during remission. These stool samples were compared to 28 controls. Active disease states were assessed using three parameters: elevated serum C-reactive protein, global physician’s assessment, and fecal calprotectin (FCP) analysis. Results demonstrated that Cyranose 320^®^ was able to successfully differentiate IBD active disease states from healthy, non-diseased patients. These results also held true when differentiating IBD remission cases from healthy individuals. In a smaller study by De Meij [64], 19 pediatric patients diagnosed with IBD (10 with UC and 9 with CD) were assessed by E-nose. Stool samples from these patients were compared to pediatric controls that did not have any known GI disease. The results showed that Cyranose 320^®^ was more aptly able to differentiate the CD cases from the controls compared to the UC cases.

Shepherd et al. [65] performed fecal headspace VOC profile analysis on healthy patients and those with IBD or irritable bowel syndrome (IBS) using GC coupled to a metal oxide sensor, a type of sensor commonly used in E-nose technology. This hybrid approach was able to differentiate IBD from IBS patients with a sensitivity of 76% and a specificity of 78%. Furthermore, differentiation of IBD patients from healthy individuals yielded a sensitivity of 79%. Similarly, Cauchi et al. [66] used GC-MS to analyze VOC profiles from patients with CD, UC, IBS, and controls. VOCs were obtained via urine, stool, serum, and breath samples, and they found that stool samples provided the best indicator for disease activity. CD was the most distinguishable condition compared to the others using GC-MS.

Other studies have utilized the Fox 4000 E-nose (MOX-based E-nose) along with FAIMS technology to assess urine VOCs from patients with IBD and healthy controls [68] Data was assessed via PCA and DFA using the E-nose, and solely by DFA while using FAIMS. PCA and DFA accuracies for the E-nose were 79.4% and 90.4%, respectively. Both modalities yielded an accuracy of over 75%, highlighting a relatively similar capability in differentiating IBD and healthy patients.

VOC analysis has had mixed reviews in its ability to discriminate between active IBD flare and a state of remission. Fecal headspace VOC analysis was performed using GC coupled to IMS (GC-IMS). Researchers classified patients diagnosed with IBD as either in an active disease state or in remission based on fecal calprotectin (FCP) levels [70]. Patients with FCP levels ≥250 mg/g were considered active, whereas patients with levels <100 mg/g were considered in remission. Results demonstrated high accuracy for differentiating both CD and UC from controls in both active and remission states, smaller differences when attempting to differentiate CD from UC, and no differences between active and remission states within each disease. Despite these poor differences, follow up studies suggested that in the active disease state group, VOC profiles were significantly different between those that were predicted to remain active and those that were predicted to enter remission (AUC 0.86) [69]. In the remission group, there was also a significant difference in the VOC profile for those that would remain in remission and those that would re-enter an active state (AUC 0.75). This study suggested that analysis of VOC profiles using GC-IMS could potentially predict disease course in patients with IBD.

Other VOC assessment modalities including SIFT-MS and FAIMS have been reported to detect differences in IBD patients. Hicks et al. [71] conducted a study to identify, quantify, and analyze VOCs emitted from patients with IBD and controls utilizing SIFT-MS. They found that certain VOCs (e.g., dimethyl sulfide, hydrogen sulfide, hydrogen cyanide, ammonia, butanal, and nonanal) were found in significant quantities in IBD-positive patients. Van Gaal et al. [72] performed a study on pediatric patients with IBD and analyzed fecal headspace VOCs using FAIMS. Results demonstrated that FAIMS was able to successfully differentiate headspace VOCs between CD profiles and controls and UC profiles and controls, but not between CD and UC cases.

Although sometimes confused with inflammatory bowel disease (Crohn’s and ulcerative colitis), irritable bowel syndrome (IBS) is a chronic, functional GI disorder characterized by abdominal pain, bloating, diarrhea (IBS-D), and/or constipation (IBS-C). While the exact pathogenesis has not been established, it is speculated that dysbiosis plays a role in the development of IBS [73]. While there are conflicting data regarding which exact bacterial species are in abundance or deficient in the setting of IBS, a well-established relationship is the proportionality of *Actinobacteria* and *Bifidobacteria* [73]. For example, multiple studies demonstrated that in a high percentage of IBS patients, there is either a significant reduction in *Bifidobacteria* or relative abundance in *Actinobacteria* [74,75,76,77,78]. One study found significantly elevated levels of serum (but not fecal) propionate and butyrate in patients with IBS-D, suggesting that these SCFAs may play a role in the pathogenesis of IBS-D [79].

Given that IBS is a functional GI disorder, researchers are struggling to significantly differentiate VOC profiles from patients with IBS and controls (Table 4). Ahmed et al. [80] analyzed fecal VOC profiles from IBS patients with predominately diarrheal symptoms (IBS-D) using GC-MS. They found the profiles from IBS patients were significantly different from those with IBD and healthy individuals, with AUCs and sensitivities all greater than 90% for each group comparison. Specifically, they found an elevated concentration in organic acids and esters from the IBS-D samples, while alternatively there was an increase in the number of aldehydes in the samples from patients with IBD.

Other studies have used multi-capillary column ion mobility spectrometry (MCC-IMS) to investigate both breath and fecal VOC samples from patients with IBS [81]. MCC-IMS is a relatively cheaper, more user-friendly version of GC-MS; however, it only allows for pseudo identification of VOCs, and therefore requires combination with other volatile analytical platforms for precise detection of compounds. MCC-IMS was able to successfully differentiate IBS from healthy individuals using both breath and fecal samples.

Given that IBS is a diagnosis of exclusion, it often requires patients to undergo invasive testing to rule out other GI diseases (e.g., IBD). Bosch et al. [82] performed a study in the pediatric population in an attempt to identify potential fecal VOC biomarkers using FAIMS. Specifically, fifteen patients with IBS, thirty patients with IBD, and thirty healthy controls were recruited, and results demonstrated an AUC of 0.94 when comparing IBS and IBD samples; however, IBS samples could not be reliably differentiated from healthy controls (AUC 0.59). Therefore, FAIMS may be a potential non-invasive method to differentiate IBS from IBD.

### 3.2. Colorectal Cancer (CRC)

Colorectal cancer is the third most common cancer among men and women and has the second highest mortality rate in cancer-related deaths worldwide. CRC is expensive to diagnose and treat; the total annual cost of CRC in the US was USD 14.1 billion, as measured in 2010 dollars [83]. In the US, the current most effective screening method for CRC is routine colonoscopy, although many individuals are reluctant to get screened. In 2015, only 6 in 10 people in the US who were eligible received a screening test [83]. This reluctance has been determined to be multifactorial, and includes, but is not limited to, the invasive nature of colonoscopy, fear of embarrassment, fear of pain and/or catching a disease during the procedure, lack of insurance, and older age [83,84]. Therefore, there is a need for a non-invasive screening test for CRC that has high sensitivity and specificity. Indeed, studies investigating potential VOC biomarkers emitted from murine colorectal adenocarcinoma cell lines using SPME and GC-MS have found significant elevations in the concentrations of 1-methoxy-hexane, 2,4-dimethyl-heptane, acetone, butylated hydroxytoluene, and many others [85,86]. Bond et al. [87] performed GC-MS fecal headspace VOC analysis from patients with CRC, adenomatous polyps, and healthy individuals, and found that a person was six times more likely to have CRC if propan-2-ol, hexan-2-one, and ethyl 3-methyl-butanoate were all present in the fecal headspace. The major findings of CRC VOC analysis are summarized in Table 5.

Arasaradnam et al. [88] assessed FAIMS ability to differentiate patients with CRC from healthy controls via urine VOC analysis, and results demonstrated a sensitivity of 88% and a specificity of 60%. The relatively higher sensitivity value was promising for screening purposes, even surpassing the values seen in fecal immunochemistry testing (FITs), which is the primary screening method in Europe [98]. Future clinical applications will likely utilize both FITs and VOC analysis for cancer screening [99]. For example, Widlak et al. [100] found a sensitivity and specificity of 80% and 93%, respectively, for detecting CRC using FITs. However, when paired with urinary VOC analysis, a new sensitivity and specificity of 97% and 72% was achieved. This study provides evidence supporting the combined use of VOC analysis with FITs to increase screening accuracy.

Batty et al. [89] analyzed fecal headspace VOCs using SIFT-MS in patients with a positive fecal occult blood test (FOBT) to determine its efficacy in classifying the stool as high-risk or low-risk for CRC. Diagnoses were confirmed with colonoscopy. They found that SIFT-MS was able to correctly classify 75% of the cases. In a study with a similar design, Mozdiak et al. [101] compared the classification capability of FAIMS and GC-IMS in patients following a FOBT and found that both FAIMS and GC-IMS were able to classify CRC patients from controls (AUCs 0.98 and 0.82, respectively) and adenomas (AUC range 0.83–0.92); however, classification of adenomas from controls was relatively weak (AUC range 0.54–0.61). However, a conflicting study found that GC-IMS was able to accurately differentiate patients with adenomas from healthy controls but not CRC [96]. In a separate study comparing GC-MS, FAIMS, and SIFT-MS in detecting CRC and polyps from healthy individuals via urine VOC analysis, GC-MS yielded the highest clinical utility, with a sensitivity and specificity of 88% and an AUC of 0.90 [93]. These studies provide evidence that following a positive FOBT, SIFT-MS, FAIMS, and/or GC-IMS could potentially be used for risk stratification of colorectal cancer.

Alustiza et al. used magnetic headspace adsorptive extraction (MAG-HSAE) followed by thermal desorption-gas chromatography-mass spectrometry (TD-GC-MS) to investigate potential VOC biomarkers from the stool samples from patients with CRC, adenomatous polyps, and healthy controls [92]. MAG-HSAE is a subtype of solid-phase extraction technique in which one end of a small neodymium magnet contains graphene oxide decorated with iron oxide magnetic nanoparticles that function as a sorbent, in which VOCs from the headspace can be directly collected and analyzed with a coupled modality (e.g., TD-GC-MS here) [102]. Using this technique, they found significantly elevated levels of p-Cresol and 3(4H)-dibenzofuranone,4a,9b-dihydro-8,9b-dimethyl- (3(4H)-DBZ) in the CRC group compared to the polyp and control groups. Additionally, they found that p-Cresol is a potential biomarker for premalignant lesions, yielding an AUC 0.69, sensitivity 83%, and specificity 63%.

E-noses and hybrid approaches using E-noses and traditional VOC analytical platforms have also been used to detect CRC. A study by Tyagi et al. used a hybrid approach of the portable E-nose 3 (PEN3), which is a QMB sensor-based E-nose, and GC coupled to time of flight-MS (GC-TOF-MS) in attempt to differentiate urinary VOCs from CRC-positive patients from controls. [94] TOF-MS has the same purpose as any MS, where particles are separated based on their m/z ratio; however, in TOF-MS, this is achieved by accelerating all particles at the same electric potential and subsequently measuring how long it takes them to reach the detector. Results were promising, with GC-TOF-MS correctly differentiating CRC patients from controls an AUC of 0.81.

De Meij et al. [97] performed Cyranose 320^®^ VOC analysis in CRC-positive patients, patients with advanced adenomas, and control patients to differentiate between their disease states. A total of 157 stool samples were collected from individuals who were undergoing an elective colonoscopy, with 60 being from CRC-positive patients, 40 from patients with AAs, and 57 from HCs (determined by no abnormalities detected during colonoscopy). Results of the study demonstrated that fecal VOC profiles differed significantly between CRC-positive patients and HCs and between AAs and HCs.

Given that symptoms of CRC can often mimic IBD, there is a need to develop non-invasive testing to distinguish these [90]. An optical and electrochemical sensor-based E-nose was used to analyze urine VOCs to differentiate patients with CRC from IBS, and results showed sensitivity and specificity values approaching 80%, highlighting E-nose technology as a potential non-invasive modality for diagnosing CRC. Van Keulen et al. [91] also performed an E-nose study (Aeonose, MOX) to detect CRC and patients with advanced adenomas (AAs) via exhaled VOC profile analysis after confirmation with colonoscopy. Results demonstrated that the Aeonose yielded a higher AUC, sensitivity, and specificity in the CRC group compared to the AA group, likely indicating exhaled VOC profiles change more as disease progresses.

### 3.3. Infectious Diarrhea

While many microorganisms are associated with the development of diarrhea, the ones that have been studied with VOC profile analysis include *Clostridioides difficile* (previously *Clostridium difficile*), *Vibrio cholera*, *Campylobacter jejuni*, and *Rotavirus*, with *C. difficile* being the most studied [57]. While sulfur-containing VOCs are strongly associated with *C. difficile*-infected stool, which gives it its characteristic odor, other molecules have also been identified, including straight- and branched-chain carboxylic acids, isocaproic acid, furan species, p-Cresol, and fluorine-containing molecules (e.g., 2-fluoro-4-methylphenol) [103,104,105,106]. Probert et al. [104] used GC-MS and found that *C. jejuni*-infected stool was shown to be associated with increased concentrations of phenols, organic acids, and indoles, while *Rotavirus*-infected stool was associated with ethyl dodecanoate, although the latter relationship was ubiquitous (Table 6) [57,104].

Garner et al. [67] used GC-MS to identify VOCs in patients with UC, *C. difficile*, *C. jejuni* infections, and healthy patients, and found hundreds of different volatile biomarkers. Because of the large quantity of VOCs identified, cluster samples for each disease state were made from selected biomarkers via discriminant analysis, resulting in a sensitivity of 96%. Additionally, the total number of VOCs decreased in UC (N = 145), *C. difficile* (N = 149), and *C. jejuni* (N = 183) compared to healthy individuals (N = 297), and it is hypothesized that this finding is likely due to the faster flow of stool through the GI tract in diseased states, and therefore there is less time for VOC production from the microbiome.

As opposed to using clusters of VOCs per disease state, Tait et al. [106] used SPME coupled to GC-MS to isolate a single volatile biomarker in patients with C. difficile infection: 2-fluoro-4-methylphenol. Although they were able to do so with a high sensitivity and specificity, the process took approximately 18 h in duration, greatly lowering its clinical utility as there are currently faster, more practical modalities for diagnosing *C. difficile* infections (e.g., E-noses and PCR testing).

Given the difficulty associated with traditional analytical systems, E-noses have potential for diagnosis of infectious colitis. McGuire et al. [107] used GC, an MOX sensor-based E-nose, and an artificial neural network (ANN) software for volatile profiling in patients with *C. difficile* infection and HCs. This method yielded a sensitivity and specificity of 85% and 80%, respectively. In a similar study by Chan et al. [108] using only Enose technology (Aeonose, specifically) coupled to an ANN, results were similar, yielding a sensitivity and specificity of 80% and 85%, respectively. These studies provide evidence in favor of using pattern recognition of VOC clusters as opposed to the individual biomarker identification seen in GC-MS.

### 3.4. Celiac Disease

Celiac disease (CelD) occurs when a susceptible individual consumes gluten and a T-cell-mediated immune reaction develops in response, resulting in small intestinal inflammation and potentially chronic malabsorption [109]. Diagnosis typically depends on serology to look for disease-specific antibodies (e.g., anti-tissue transglutaminase IgM antibodies) and duodenal biopsy to look for intraepithelial lymphocytosis, villous atrophy, and crypt hyperplasia [110]. The use of headspace VOCs as a potential diagnostic modality is an area of increasing research, as this would eliminate the need for the current invasive methods. A pilot study conducted by Di Cagno et al. [111] showed that gluten-free diets in children with diagnosed CelD resulted in modulation of the microbiota and subsequent VOC profiles, which was analyzed with bacterial 16S-DNA sequencing and GC-MS-SPME, respectively. This study demonstrated that dietary intake may not only impact the microbiota, but also the headspace VOC profiles. Indeed, McFarlane et al. [112] used both FAIMS and GC-TOF-MS to analyze urinary VOC profiles from patients with CelD on a gluten-free diet, patients with CelD consuming 3 g of gluten per day, and HCs, and found that minimal gluten exposure to patients with CelD resulted in significant VOC profile changes. Specifically, six VOCs were persistently altered for 2 weeks post-exposure, with one VOC (N-methyltaurine) remaining altered after the 2-week mark. Table 7 summarizes the studies using VOC profiles to diagnose CelD.

Rouvroye et al. [113] performed GC-IMS fecal headspace VOC analysis in patients with CelD on a gluten-free diet, refractory CelD (RCD), and HCs. Results showed a significant VOC profile difference between the CelD and RCD groups as well as the CelD and HC groups. However, there were no significant differences seen between the RCD and HC groups (*p* = 0.310). This study is limited by the relatively low number of participants (30 total), but provides evidence in favor of using VOC profiles as a potential non-invasive diagnostic modality for RCD.

CelD can often be mistaken for IBS, given that symptoms often overlap and can be difficult to differentiate without invasive testing. To identify a potential non-invasive method, Arasaradnam et al. [114] performed FAIMS urinary VOC analysis in patients with CelD and IBS-D and found that it was able to differentiate them with high accuracy, sensitivity, and specificity. Additionally, GC-MS was performed on fecal samples to identify potential VOC biomarkers, and they found that 1,3,5,7-cyclooctatetraene was only found associated with CelD and not IBS-D. This study provides evidence in favor of using FAIMS to differentiate CelD from IBS-D and that the presence of 1,3,5,7-cyclooctatetraene in CelD specimens requires further validation.

### 3.5. Late-Onset Sepsis and Necrotizing Enterocolitis

While it is important to detect VOC profile differences in already-diseased individuals from controls, a far more clinically useful application would be predicting disease onset. Two diseases that show promise thus far are neonatal late-onset sepsis and necrotizing enterocolitis. Neonatal late-onset sepsis (LOS) is defined as sepsis that occurs at least 72 h after birth. In developed and developing countries, coagulase-negative *Staphylococci* (CONS) spp. are the leading cause of LOS, accounting for 53.7–77.7% and 35.5–47.4% of cases, respectively [115]. Following CONS, in the United States, the most frequently reported LOS-causing pathogens include *S. aureus*, *Candida* spp., *E. coli, Klebsiella* spp., and *Enterobacter* spp., with Gram-positive organisms being more common, but Gram-negative organisms having a higher mortality rate [115]. LOS primarily affects preterm infants, having incidence rates that vary anywhere from 20 to 38% in the first 4 months of life and a mortality rate between 13 and 19% [116]. This makes LOS one of the leading causes of death in neonatal intensive care units (NICUs). Infants that do survive LOS are at an increased risk for long-term effects, including neurodevelopmental delay (i.e., cerebral palsy, vision impairment, poor psychomotor skills, impaired head growth), growth abnormalities, intestinal and respiratory infections, and others [116,117]. While there are screening options for LOS, they often involve invasive procedures, which ironically increase the risk of developing LOS independently [118]. Central venous catheters (CVCs) have been attributed to being a major source of LOS, although studies have demonstrated that the organisms isolated from blood cultures do not perfectly match genetically with the bacteria cultured from the catheters [119,120]. Instead, there is a higher genetic similarity between the cultured LOS pathogen and isolates from the GI tract, indicating that the gut is a potential origin of these pathogens [121]. Using the notion that dysbiosis precedes LOS, studies have shown that isolated pathogens from blood cultures can be detected in the GI tract several days before the onset of clinical symptoms (Table 8) [122,123,124,125].

Berkhout et al. [126] performed a prospective, multicenter cohort study to analyze fecal headspace VOCs from preterm infants born at a gestational age (GA) ≤ 30 weeks. Fecal samples from preterm infants were collected daily from the NICU up until a postnatal age of 28 days old. A total of 843 preterm infants were included, with 127 cases of LOS and 127 matched controls. The most frequent causative pathogens included *E. coli*, *Staph aureus*, and *Staph epidermidis*. FAIMS was able to significantly differentiate fecal VOCs 3 days (t_-3_), 2 days (t_-2_), and 1 day (t_-1_) preceding the onset of clinical symptoms for all three pathogens.

As discussed earlier, there is evidence suggesting a higher genetic similarity between pathogens that cause LOS and isolates from the GI tract rather than isolates from blood cultures associated with CVCs. In a similar methodology as the previous study described, Berkhout et al. [127] used FAIMS to analyze fecal headspace VOCs from preterm infants with LOS without a CVC and HCs. FAIMS was able to significantly differentiate VOC profiles at t_-3_ and t_-1_, but not at t_-2_ (*p* = 0.061). This study provides further evidence supporting aberrations in the gut microbiota may play a role in the pathogenesis of LOS.

Frerichs et al. [128] Previous studies using GC-IMS to predict preclinical onset of neonatal LOS found that fecal VOC profiles were most profoundly different at two days prior to clinical onset (t_-2_) and one day prior to clinical onset (t_-1_) for *E. coli* LOS and at three days prior to clinical onset (t_-3_) and t_-2_ for all Gram-negative LOS. Additionally, GC-TOF-MS was used to identify unique metabolites. For proper discrimination of Gram-negative LOS from HCs, ethyl acetate, ethyl 2-(methylamino)acetate, ethyl 2-hydroxypropanoate, prop-1-ene, butane-2,3-dione, and 2,2,4,4-tetramethylpentane were found to be most important, while heptanal was found to be the most important for the discrimination of CONS-LOS. Fecal VOC profiles of 36 infants with LOS were compared to 40 matched controls using Cyranose 320^®^ [121]. Results showed that the Cyranose 320^®^ was able to discriminate between LOS-positive infants from the controls 3 days, 2 days, and 1 day before the clinical onset of LOS. However, the E-nose was unable to differentiate between LOS-positive infants from controls beyond 3 days prior to LOS onset.

Necrotizing Enterocolitis (NEC) is a gastrointestinal emergency primarily affecting preterm, formula-fed infants, and is characterized by intestinal inflammation that can result in necrosis and perforation of the bowel, sepsis, and death. The total annual cost in the US is estimated to be between USD 500 million and USD 1 billion, largely due to the current diagnostic methods used, prolonged hospital admission required for treatment, and the possible need for surgery [129]. While the pathophysiology of NEC is not completely understood, there are established risk factors, including preterm delivery, low birth weight, formula-feeding as opposed to breastfeeding, and dysbiosis [130]. In 2009, Garner et al. [131] performed a pilot study investigating potential VOC biomarkers associated with NEC via SPME coupled to GC-MS. Results of the study demonstrated a decreased total number of VOCs along with an absence of 2-ethylhexyl acetic ester, decanoic acid ethyl ester, dodecanoic acid ethyl ester, and hexadecanoic acid ethyl ester in patients with NEC compared to controls, suggesting VOC profiles could potentially be used in screening for NEC. Indeed, Probert et al. [132] used SPME coupled to GC-MS to identify potential fecal VOC biomarkers associated with the onset of NEC, and found a strong prediction accuracy (AUC 0.75–0.76) associated with (Z)-hept-2-enal, pent-1-en-3-one, 2-ethylfuran, pentanal, and 2-pentylfuran up to 3–4 days prior to clinical onset. Furthermore, Hosfield et al. [133] performed 16S rRNA microbial sequencing and fecal VOC analysis of mouse pups with with NEC and breastfed controls using Cyranose 320^®^ and found a significant abundance of *Lactobacillus* and decreased concentration of *E. coli* in the HCs compared to the pups with NEC (*p* < 0.05).

Diagnosis of NEC relies primarily on abdominal X-rays to detect the presence of air in the intestines, portal vein of the liver, and/or peritoneum as well as other diagnostic and clinical markers [134]. Not only are X-rays expensive and expose the infant to radiation, but they are also not ordered until clinical symptoms of NEC are exhibited, often when the disease has already significantly progressed. Thus, VOC analysis provides a potential solution for earlier detection of NEC (Table 9).

De Meij et al. [135] assessed VOCs from frozen stool samples of 128 preterm neonates who had NEC, sepsis, or were considered healthy controls [84]. VOC profile analysis of these stool samples was performed with Cyranose 320^®^. Fecal samples were clustered into three time windows: 5 and 4 days before diagnosis (t_-5_,_-4_), 3 and 2 days before diagnosis (t_-3_,_-2_), and 1 day before and the day of diagnosis (t_-1_,_0_). Results demonstrated that the E-nose was able to accurately discriminate the VOC profiles between infants with NEC from the controls at both t_-3_,_-2_ and at t_-1_,_0_. The accuracy of the VOC profile analysis increased as time approached NEC clinical onset. Additionally, NEC VOC profiles were significantly differentiated from the VOC profiles of the infants with sepsis at t_-3_,_-2_ but not at t_-1_,_0_. This study supports the use of E-noses as a screening test for NEC from 2–3 days before the onset of symptoms.

## 4. Conclusions

Intestinal diseases such as colorectal cancer, inflammatory bowel disease, and necrotizing enterocolitis impact a large number of patients annually. These diseases are often costly and impact a patient’s quality of life. The use of volatile analytical platforms and their role in non-invasively diagnosing GI diseases are areas of increasing research and clinical application. Despite their ability to detect various GI illnesses via VOC analysis, many of the discussed analytical platforms (e.g., GC-MS, FAIMS, and GC-IMS) are unlikely to be implemented into healthcare settings due to their cost, size, time inefficiency, and difficulty of use. Instead, they are more likely to be used clinically in conjunction with E-noses. E-noses are exciting novel technologies to the healthcare industry and can hopefully be implemented into clinical settings in the future.

## Figures and Tables

**Table 1:** Pros and Cons of Various Volatile Analytical Platforms Used for Detecting GI Diseases.

Volatile Analytical Platform	Pros	Cons
E-Nose	Non-invasive detection method Portable and easy to use Can be used for real-time monitoring	Requires calibration with known standards Limited ability to identify specific VOCs May require regular maintenance and sensor replacement
GC-MS	High sensitivity and selectivity Ability to identify specific VOCs Quantitative analysis Established technique with extensive database of volatile compounds for comparison	Complex instrumentation and technical expertise required Time-consuming sample preparation Extensive equipment and operational costs Laboratory-based setup, not suitable for point-of-care testing
IMS	Rapid analysis Portable and hand-held devices available Real-time monitoring capability Can detect VOCs at low concentrations	Lower sensitivity compared to GC-MS Difficulty in identifying and quantifying specific VOCs due to overlapping spectra Limited database of volatile compounds for comparison
FAIMS	High selectivity Rapid analysis Non-invasive detection method Some portable and hand-held devices available Lower costs Less complex sample preparation	Requires careful preparation of instrument parameters for optimal separation and sensitivity Limited capacity for high-throughput analysis Requires the development and expansion of comprehensive databases for accurate analysis Regulatory and validation challenges need to be addressed for clinical implementation
SIFT-MS	Real-time analysis High sensitivity Wide range of detectable compounds No sample preparation necessary for most models	Expensive instrumentation and maintenance costs Sample dilution may be necessary due to limited upper limit for highly concentration samples Sensitivity may be limited for complex or certain classes of molecules

A general overview of these volatile analytical platforms, and the specific advantages and disadvantages may vary depending on the specific models and implementations of them.

**Table 2:** Comparison of Different E-nose Sensors.

Sensor Type	Sensitive Material	Detection Principle	Advantages	Disadvantages
Conducting Polymer	Modified conducting polymers	Change in resistance	Sensitive to many VOCs, inexpensive, operates at ambient temperature, fast response time	Affected by temperature and humidity, limited sensor life
Optical	Photodiode, sensitive to light	Optical changes and light modulation	High sensitivity, has potential to identify individual compounds in large mixtures	More expensive to operate, low portability potential
Surface Acoustic Wave (SAW)	Organic/inorganic film layers	Mass change (frequency shift)	Easily portable, inexpensive, high sensitivity to essentially all gases	Affected by temperature, type of sensor coating used impacts specificity to certain analyte groups
Quartz Crystal Microbalance (QMB)	Organic/inorganic film layers	Mass change (frequency shift)	High sensitivity and precision, multiple sensor coatings	Signal-to-noise ratio poor, affected by temperature and humidity
Calorimetric or Catalytic Bead (CB)	Pellistor	Temperature or heat change from chemical reactions	Quick recovery and response time, particularly high specificity for oxidized compounds	Requires high temperatures for proper operation, sensitive only to compounds containing oxygen
Electrochemical (EC)	Solid/liquid electrolytes	Current/voltage changes	Operates at ambient temperature, high sensitivity to electrochemically active VOCs, requires little power for use	Bulky size negatively impacts portability potential, simple and low molecular weight gases have limited sensitivity
Catalytic Field-Effect (MOSFET)	Catalytic metals	Electric field change	Inexpensive, small	Requires strict environmental conditions for operation, sensitivity low to ammonia and carbon dioxide
Semi-Conducting Metal Oxides (MOX)	Doped semi-conducting metal oxides	Change in resistance	High sensitivity, rapid response, and recovery times for low molecular weight compounds	Requires high temperatures for proper operation, sensitive to humidity, poor precision

**Table 3:** Diagnosis of IBD with Volatile Analytical Platforms.

Disease: Control	Volatile Analytical Platform	Sensitivity (%)	Specificity (%)	AUC	Sample No: Control No	References
CD, active: HC	Cyranose 320^®^	86	67	0.85	29:28	[63]
CD, remission: HC		94	94	0.94	29:28	[63]
UC, active: HC		100	100	1.00	26:28	[63]
UC, remission: HC		94	94	0.94	26:28	[63]
CD, active: UC, active		97	92	0.96	29:26	[63]
CD, remission: UC, remission		88	72	0.81	29:26	[63]
CD: Control	Cyranose 320^®^	92	100	0.98	9:10	[64]
UC: HC		75	77	0.75	10:10	[64]
IBD:IBS	GC-MOX	76	78	-	102:135	[65]
IBD: HC		79	-	-	135:138	[65]
CD: HC	GC-MS	93	78	0.97	24:20	[66]
UC: HC		43	69	0.54	19:20	[66]
UC: HC	GC-MS	96	-	-	18:30	[67]
IBD: HC	Fox 4000 and FAIMS	-	-	0.85	48:14	[68]
IBD, active	GC-IMS	-	-	0.86	37	[69]
IBD, remission		-	-	0.75	49	[69]
CD, active: HC	GC-IMS	-	-	0.96	107:227	[70]
CD, remission: HC		-	-	0.95	84:227	[70]
UC, active: HC		-	-	0.96	80:227	[70]
UC, remission: HC		-	-	0.95	63:227	[70]
UC: CD		-	-	0.55	143:191	[70]
UC, active: UC, remission		-	-	0.63	80:63	[70]
CD, active: CD, remission		-	-	0.52	107:84	[70]
CD: HC	SIFT-MS	-	-	0.86	18:18	[71]
UC: HC		-	-	0.74	20:18	[71]
CD: HC		-	-	0.83	18:20	[71]
CD: HC	FAIMS	83	83	0.76	23:24	[72]
UC: HC		77	75	0.74	13:24	[72]
CD: UC		65	62	0.67	23:13	[72]
Pooled Mean	-	84	82	0.82	-	-

CD = Crohn’s disease; UC = ulcerative colitis; HC = healthy control; IBD = inflammatory bowel disease; IBS = irritable bowel syndrome.

**Table 4:** Diagnosis of IBS with Volatile Analytical Platforms.

Disease: Control	Volatile Analytical Platform	Sensitivity (%)	Specificity (%)	AUC	Sample No: Control No	References
IBS: HC	GC-MOX	54	-	-	104:137	[65]
IBS: HC	GC-MS	51	71	0.63	28:20	[66]
IBS-D: HC	GC-MS	90	80	0.94	30:109	[80]
IBS-D: IBD, active		96	80	0.98	30:110	[80]
IBS-D: CD		94	82	0.97	62:30	[80]
IBS-D: UC		96	80	0.96	48:30	[80]
IBS (breath): HC	MCC-IMS	-	-	0.62	72:24	[81]
IBS (fecal): HC		-	-	0.80	62:19	[81]
IBS: IBD	FAIMS	100	87	0.94	15:30	[82]
IBS: HC		60	63	0.59	15:30	[82]
Pooled Mean	-	80	78	0.83	-	-

IBS = irritable bowel syndrome; UC = ulcerative colitis; IBD = inflammatory bowel disease; HC = healthy control; IBS-D = irritable bowel syndrome, diarrheal predominant.

**Table 5:** Diagnosis of CRC with Volatile Analytical Platforms.

Disease: Control	Volatile Analytical Platform	Sensitivity (%)	Specificity (%)	AUC	Disease No: Control No	References
CRC: HC	FAIMS	88	60	-	83:50	[88]
High-Risk CRC: Low-Risk CRC	SIFT-MS	72	78	-	31:31	[89]
CRC: IBS	Enose (optical + electrochemical sensor)	78	79	-	-	[90]
CRC: HC	Enose (MOX)	95	64	0.84	70:125	[91]
AA: HC		79	59	0.73	117:125	[91]
CRC (p-Cresol): Adenomatous Polyps + HC	MAG-HSAE-TD-GC-MS	83	82	0.85	24:56	[92]
CRC ((3(4H)-DBZ): Adenomatous Polyps + HC		78	75	0.77	24:56	[92]
CRC (p-Cresol + 3(4H)-DBZ): Adenomatous Polyps + HCs		87	79	0.86	24:56	[92]
CRC + Polyps: HCs	GC-MS	88	88	0.90	109:115	[93]
CRC: HC	Enose + GC-TOF-MS	-	-	0.81	58:38	[94]
CRC + Stomach Cancer: HC	GC	96	91	-	29:16	[95]
CRC + Stomach Cancer (+Lipid Peroxidation Products): HC		100	100	-	29:16	[95]
CRC: HC	GC-IMS	100	100	0.96	14:227	[96]
AA:HC		97	94	0.96	64:227	[96]
LA: HC		99	91	0.96	69:227	[96]
SA: HC		96	93	0.96	127:227	[96]
CRC: Adenomas		68	39	0.45	14:260	[96]
AA: HC	Cyranose 320^®^	62	86	0.79	60:57	[97]
CRC: HC		85	87	0.92	40:57	[97]
CRC: AA		75	73	0.92	40:47	[97]
CRC + AA: HC		85	68	0.92	40:57	[97]
Pooled Mean	-	81	79	0.85	-	-

CRC = colorectal cancer; HC = healthy control; AA = advanced adenoma; LA = large adenoma; SA = small adenoma; IBS = irritable bowel syndrome.

**Table 6:** Diagnosis of Infectious Diarrhea with Volatile Analytical Platforms.

Disease: Control	Volatile Analytical Platform	Sensitivity (%)	Specificity (%)	AUC	Disease No: Control No	References
*C. difficile*: HC	GC-MS	83	97	-	6:6	[104]
*C. Jejuni*: HC		100	92	-	5:6	[104]
*Rotavirus*: HC		100	97	-	5:6	[104]
Non-*Rotavirus*: HC		63	96	-	19:6	[104]
*C. difficile*: HC	GC-MS	96	-	-	22:30	[67]
*C. Jejuni*: HC		96	-	-	31:30	[67]
*C. difficile: HC*	SPME-GC-MS	83	100	-	77:23	[106]
*C. difficile:* HC	GC + Enose	85	80	-	50:50	[107]
*C. difficile:* HC	Enose	80	85	0.84	20:53	[108]
Pooled Mean	-	87	92	0.84	-	-

HC = healthy control.

**Table 7:** Diagnosis of Celiac Disease with Volatile Analytical Platforms.

Disease: Control	Volatile Analytical Platform	Sensitivity (%)	Specificity (%)	AUC	Disease No: Control No	References
CelD: RCD	GC-IMS	85	86	0.91	13:7	[113]
CelD: HC		92	65	0.71	13:10	[113]
RCD: HC		71	57	0.57	7:10	[113]
CelD: IBS-D	FAIMS	85	85	0.91	27:20	[114]
Pooled Mean	-	83	73	0.78	-	-

CelD = celiac disease; RCD = refractory celiac disease; HC = healthy control; IBS-D = irritable bowel syndrome, diarrheal predominant.

**Table 8:** Diagnosis of Late-Onset Sepsis Using Volatile Analytical Platforms.

Disease: Control	Volatile Analytical Platform	Sensitivity (%)	Specificity (%)	AUC	Disease No: Control No	References
*E. coli* (t_-3_): HC	FAIMS	91	82	0.88	11:11	[126]
*E. coli* (t_-2_): HC		100	89	0.99	9:9	[126]
*E. coli* (t_-1_): HC		92	77	0.86	13:13	[126]
*S. aureus* (t_-3_): HC		88	81	0.85	16:16	[126]
*S. aureus* (t_-2_): HC		85	62	0.70	13:13	[126]
*S. aureus* (t_-1_): HC		73	80	0.80	15:15	[126]
*S. epidermidis* (t_-3_): HC		84	89	0.90	19:19	[126]
*S. epidermidis* (t_-2_): HC		82	68	0.78	22:22	[126]
*S. epidermidis* (t_-1_): HC		54	71	0.63	35:35	[126]
CVC-negative LOS (t_-3_): HC	FAIMS	82	65	0.78	17:17	[127]
CVC-negative LOS (t_-2_): HC		59	82	0.65	17:17	[127]
CVC-negative LOS (t_-1_): HC		61	83	0.78	18:18	[127]
*E. coli* (t_-2_): HC	GC-IMS	92	64	0.83	12:14	[128]
E. coli (t_-1_): HC		64	77	0.73	14:13	[128]
Gram-negative (t_-3_): HC		88	74	0.85	24:20	[128]
Gram-negative (t_-2_): HC		65	83	0.81	23:29	[128]
LOS (t_-3_): HC	Cyranose 320^®^	57	62	0.70	36:40	[121]
LOS (t_-2_): HC		75	71	0.78	36:40	[121]
LOS (t_-1_): HC		64	64	0.70	36:40	[121]
Pooled Mean	-	77	75	0.88	-	-

HC = healthy control; CVC = central venous catheter; LOS = late-onset sepsis.

**Table 9:** Diagnosis of Necrotizing Enterocolitis with E-Nose Technology.

Disease: Control	Volatile Analytical Platform	Sensitivity (%)	Specificity (%)	AUC	Disease No: Control No	References
NEC (t_-3_,_-2_): HC	Cyranose 320^®^	83	75	0.77	13:84	[135]
NEC (t_-1_,_0_): HC		89	89	0.99	13:84	[135]
NEC (t_-3_,_-2_): sepsis		83	75	0.80	13:31	[135]
NEC (t_-1_,_0_): sepsis		89	57	0.64	13:31	[135]
Pooled Mean	-	86	74	0.80	-	-

NEC = necrotizing enterocolitis; HC = healthy controls.

## Data Availability

Not applicable.
